# Nervous Aphonia Cured by Inhalation of Chloroform

**Published:** 1876-06

**Authors:** 


Nervous Aphonia Cured by Inhalation of Chloroform.—Dr. de Ridder, of Liege, reports a case in which these inhalations produced the happiest results. The patient, a woman of 28 years of age, of a good constitution, became aphonic while suffering from an ordinary cold. This aphonia resisted all the remedies employed, after the disappearance of the inflammatory symptoms. As the patient was hysterical, M. de Ridder, thinking that the aphonia was nervous, employed without success assafœtida pills, and then a narcotico anti-spasmodic mixture. Finally he made her inhale the vapor of chloroform for one or two minutes every hour, in such a manner as not to produce anæsthesia, but just enough to produce a little giddiness or stupefaction. The aphonia yielded little by little, and at the end of two days had completely disappeared. Five months afterwards chloroform was again employed, with like success, for a recurrence of the attack. Finally, a year later, the same aphonia, accompanied by a spasmodic hiccough and incessant vomiting of a glairy matter, showed itself after a violent attack of hysteria. The employment of chloroform, administered in the same way, again succeeded as well as before. The hiccough and the vomiting yielded on the employment of ether.—*Journal de Med. et de Chir.*

